# A 14-Bit, 12 V-to-100 V Voltage Compliance Electrical Stimulator with Redundant Digital Calibration

**DOI:** 10.3390/mi14112001

**Published:** 2023-10-28

**Authors:** Kangyu Su, Zhang Qiu, Jian Xu

**Affiliations:** 1 College of Information and Electronics Engineering, Zhejiang University, Hangzhou 310027, China; 3170105164@zju.edu.cn (K.S.); qz_gd@zju.edu.cn (Z.Q.); 2 MOE Frontier Science Center for Brain Science and Brain-Machine Integration, Zhejiang University, Hangzhou 310058, China; 3 Nanhu Brain-Computer Interface Institute, Hangzhou 311100, China; 4 Affiliated Mental Health Center & Hangzhou Seventh People’s Hospital, Zhejiang University School of Medicine, Hangzhou 310013, China

**Keywords:** adaptive supply voltage, high voltage, high-precision current, flexible waveform, neurostimulator, electrical stimulator

## Abstract

Electrical stimulation is an important technique for modulating the functions of the nervous system through electrical stimulus. To implement a more competitive prototype that can tackle the domain-specific difficulties of existing electrical stimulators, three key techniques are proposed in this work. Firstly, a load-adaptive power saving technique called over-voltage detection is implemented to automatically adjust the supply voltage. Secondly, redundant digital calibration (RDC) is proposed to improve current accuracy and ensure safety during long-term electrical stimulation without costing too much circuit area and power. Thirdly, a flexible waveform generator is designed to provide arbitrary stimulus waveforms for particular applications. Measurement results show the stimulator can adjust the supply voltage from 12 V to 100 V automatically, and the measured effective resolution of the stimulation current reaches 14 bits in a full range of 6.5 mA. Without applying charge balancing techniques, the average mismatch between the cathodic and anodic current pulses in biphasic stimulus is 0.0427%. The proposed electrical stimulator can generate arbitrary stimulus waveforms, including sine, triangle, rectangle, etc., and it is supposed to be competitive for implantable and wearable devices.

## 1. Introduction

Electrical neuromodulation is an effective treatment for loss of function due to injury or disease, and it has been widely used in both implantable and wearable devices, including deep brain stimulation (DBS) [1,2,3,4], spinal cord stimulation (SCS) [5,6,7,8], peripheral neural stimulation (PNS) [9,10,11], muscle stimulation [12,13,14], and neural prosthetics [15,16,17]. Electrical stimulators can be divided into voltage-controlled stimulators (VCSs) [18,19], switched-capacitor stimulators (SCSs) [1,20,21], and current-controlled stimulators (CCSs) [17,22,23,24,25]. Among them, CCS is widely used due to its safety features. For example, the total amount of charge can be easily controlled by adjusting stimulation current amplitude and pulse width [26].

Significant research efforts have been made in CCS over the past few decades, but the majority of existing CCS structures are restricted to specific scenarios. The design of a more competitive electrical stimulator faces three major challenges. (1) Fixed supply voltage. Adaptive supply voltages are needed to achieve the desired power efficiency [27,28,29]. (2) Potential safety issues. Charge imbalance will lead to cell calcification and cause safety issues [30,31]. (3) Simple waveform configuration. Rectangular pulse is difficult for some applications to deliver optimal stimulation [32,33].

**Figure 1:**
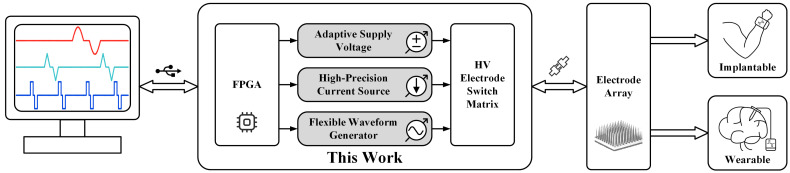
Conceptual structures of the proposed competitive electrical stimulator, which is controlled by a computer via USB and can be used for both implantable and wearable devices.

To overcome the aforementioned issues, a competitive CCS prototype based on commercial discrete components and a field programmable gate array (FPGA) is presented in this paper, and three key techniques are developed to optimize the system’s performance. (1) Adaptive supply voltage. An over-voltage detection algorithm for adaptive voltage regulation is proposed, where the designed prototype can automatically adjust the output voltage from 12 V to 100 V in different scenarios through a feedback loop consisting of a digital-to-analog converter (DAC) and an analog-to-digital converter (ADC). (2) High-precision current source. Two 12-bit DACs with RDC are used to achieve a 14-bit output current of up to 6.5 mA. By combining this technique with an H-bridge stimulator structure, superior charge balancing performance can be achieved. (3) Flexible waveform generator. A finite state machine (FSM) is developed to control the two 12-bit DACs in real time when stimulation begins, thus producing arbitrary waveforms with a temporal accuracy of 1.6 μS. Figure 1 shows the conceptual structures of the proposed prototype based on these three techniques, which is controlled by a computer via a universal serial bus (USB) and connected to an electrode array for implantable and wearable applications.

The rest of this paper is organized as follows. Section 2 presents an overview of current works in the literature. Section 3 describes the system architecture and circuit implementation. The circuit measurement results are given in Section 4. Finally, Section 5 gives a conclusion on this paper and discusses future work.

## 2. Overview of Current Works

Designing a competitive electrical stimulator requires comprehensive considerations of efficiency, safety, performance, and other factors [23,24,25,34]. In the following subsections, a brief overview of the current works addressing the aforementioned challenges is presented.

### 2.1. Improvement for Fixed Supply Voltage

Electrical stimulators have varied electrode impedance (from 1.5 kΩ to 1 MΩ) and current requirements (from 50 μA to 20 mA) depending on the specific applications [35,36,37,38,39]. For example, it has been shown that electrical stimulators used in retinal implants need a stimulation current of 50 μA to generate image signals [24]. In contrast, a much higher current of up to 20 mA is required to achieve the desired neuromodulation effects in muscle stimulation [31]. Circuit structures with a fixed supply voltage are relatively simple to implement but cannot output sufficient current in scenarios with high electrode impedance. Furthermore, significant power waste is unavoidable in low electrode impedance applications. Programmable supply voltage structures cannot adjust the supply voltage automatically and require external intervention under different impedance environments [22,28]. Some adaptive supply voltage designs can partly solve this problem, but their voltage adjustment range is limited [22,40].

### 2.2. Improvement for Potential Safety Issues

Calibrating the mismatch between anodic and cathodic pulses decreases the probability of electrode oxidation and cell calcification, which will improve the safety of the electrical stimulator during long-term stimulation. However, achieving perfect charge balance is theoretically impossible. A residual average direct current (DC) of higher than 100 nA is closely associated with tissue damage, making it an important threshold for avoiding charge imbalance [41]. Several methods for achieving charge balance include blocking capacitor, passive charge balance, and active charge balance. Blocking capacitor is not a general solution for scenarios with a large number of stimulation channels, as it significantly increases the circuit area. Passive charge balance redistributes the residual charge with a simple circuit structure [42]. However, the time for reestablishing balance depends on the RC time constant of the electrode–tissue interface, which will limit the stimulation rate. Active charge balance is a technique that monitors the voltage of electrodes and adjusts the charge accordingly when the voltage exceeds a safe limit. This technique improves the charge balance performance but also adds significant complexity to the circuit structure. Additionally, improving the current accuracy is a possible solution, but it needs to overcome difficulties in circuit design and calibration algorithm development [17].

### 2.3. Improvement for Simple Waveform Configuration

With the development of neuroscience, optimal stimulus pulse shapes have become a popular research topic. None of the waveforms satisfies the energy, charge, and power optimization simultaneously [32]. Asymmetric stimulus waveforms can help reduce crosstalk between different channels and prevent excitation during discharge [43]. Gaussian, linear, exponential, and sinusoidal waveforms are a few examples of non-rectangular shapes that have been demonstrated to have strong stimulating effects [44]. Several designs have shown that arbitrary stimulus waveforms are required for achieving optimal results in different applications [22,43,45]. A large number of CCS are based on rectangular stimulus and have relatively simple circuit architectures. However, they only offer basic waveforms and cannot verify any potential optimal options. Recent systems with real-time waveform control capabilities [40,46,47] are limited in current accuracy, which makes it difficult to generate flexible waveforms.

## 3. System Architecture and Circuit Implementation

**Figure 2:**
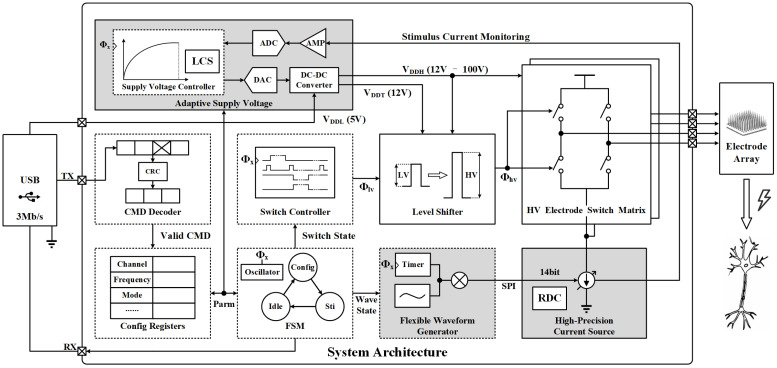
Simplified block diagrams of the proposed electrical stimulator based on commercial components with three key techniques, including adaptive supply voltage, high-precision current source, and flexible waveform generator. $V_{DDL}$ is the input voltage from USB, $V_{DDT}$ is the supply voltage for level shifter, and $V_{DDH}$ is the adaptive supply voltage for the HV electrode matrix. $\Phi_{x}$ is the clock signal, and $\Phi_{lv}$ is the LV switch control signal boosted by the level shifter to generate $\Phi_{hv}$.

Figure 2 shows the block diagrams of the proposed prototype based on commercial discrete components, including a low-power FPGA, an ADC, three DACs, an operational amplifier (OPAMP), two DC-DC converters, eight high-voltage (HV) complementary metal-oxide-semiconductor transistors (CMOS), four HV level shifters, four phototransistors, a USB interface, and an electrode connector. The prototype is connected to a USB interface to obtain a 5 V supply voltage ($V_{DDL}$) and communicates with the computer via the universal asynchronous receiver/transmitter (UART) protocol at 3 Mb/s. An FPGA is employed to implement all digital circuits consisting of a supply voltage controller, a command (CMD) decoder, a switch controller, config registers, an FSM, and a flexible waveform generator. The output of the prototype is connected to an electrode array, which delivers stimulus to the target area for performing effective neuromodulation. Table 1 summarizes the commercial discrete components used in this prototype. Next, each block is presented in detail.

### 3.1. Adaptive Supply Voltage

The minimum supply voltage $V_{DDHmin}$ of the electrical stimulator is calculated as

(1) $$V_{DDHmin}=I_{stim}\times R_{load}+\frac{I_{stim}\times T_{stim}}{C_{load}}+V_{h},$$

where $I_{stim}$ is the amplitude of the stimulation current, $R_{load}$ and $C_{load}$ represent the resistive and capacitive components, respectively, $T_{stim}$ is the maximum stimulation period, and $V_{h}$ is the headroom voltage required for the circuit. The stimulation current $I_{stim}$ depends on the value of either $V_{DDH}$ or $V_{DDHmin}$. $I_{stim}$ is determined by $V_{DDH}$ if $V_{DDH}$ falls below $V_{DDHmin}$. Conversely, when $V_{DDH}$ exceeds $V_{DDHmin}$, $I_{stim}$ is constant and equal to the output of the high-precision current source block.

**Figure 3:**
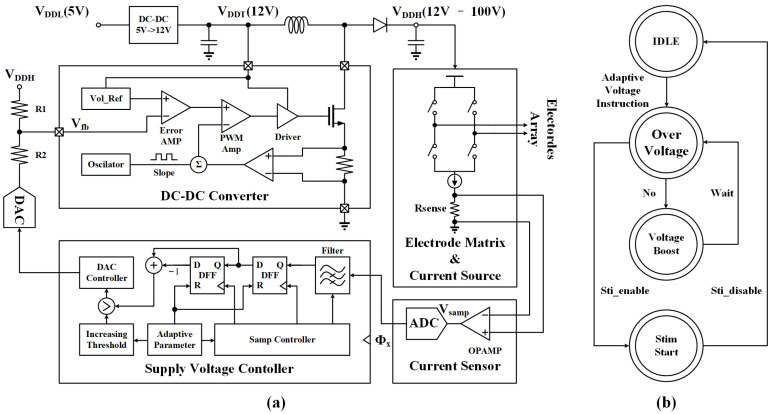
(**a**) Circuit implementation of the designed adaptive supply voltage block based on the proposed over-voltage detection algorithm. $V_{DDL}$ is the input voltage from USB, $V_{DDT}$ is the supply voltage for driver, and $V_{DDH}$ is the adaptive supply voltage for HV electrode matrix. $V_{samp}$ is linearly related to the stimulation current. Supply voltage controller processes filtered $V_{samp}$ and modulates DAC to control $V_{DDH}$. (**b**) State transition diagram of the over-voltage detection algorithm.

As shown in Figure 3, there is a feedback loop in the DC-DC converter. The comparison results of $V_{fb}$ and $V_{ref}$ are sent to a pulse width modulation amplifier (PWM Amp) for controlling the $V_{DDH}$, which ensures that the output voltage satisfies

(2) $$V_{DDH}=V_{fb}\times \frac{R_{1}+R_{2}}{R_{2}}-V_{dac}\times \frac{R_{1}}{R_{2}},$$

where $R_{1}$ and $R_{2}$ are the divider resistances, $V_{dac}$ presents the DAC output voltage, and $V_{fb}$ is the feedback voltage of the DC-DC converter.

In the prototype, $V_{DDH}$ is controlled by adjusting $V_{dac}$, and $V_{DDH}$ should be set to $V_{DDHmin}$ to achieve the best efficiency during stimulation. To find the optimal $V_{DDH}$, a novel algorithm called over-voltage detection is presented in this paper. The proposed algorithm does not require an arithmetic–logical unit (ALU) to calculate the electrode impedance and appropriate $V_{DDH}$. The key steps of the algorithm are given as follows. Firstly, the algorithm sets the desired value of $I_{stim}$ and starts the adaptive supply voltage modulation process. Secondly, $V_{DDH}$ is adjusted to its lowest possible value of 12 V since the impedance of the electrode is unknown. Thirdly, a series of rectangular waveforms is generated for prestimulation. At the same time, an ADC is used to sample $V_{samp}$ at a frequency of 10 KHz, which is proportional to $I_{stim}$. Additionally, a 64-order filter is used to process $V_{samp}$ to suppress noise, and the supply voltage controller constantly monitors processed $V_{samp}$ and increases $V_{DDH}$ gradually. Finally, the over-voltage detection algorithm identifies whether the prototype enters the over-voltage phase when $V_{samp}$ stops increasing or its increase rate slows down. Then, it stops sampling $V_{samp}$ and maintains the current value of $V_{DDH}$. To avoid double-counting, the optimal value of $V_{DDH}$ is stored in the DAC controller.

According to the algorithm with two DC-DC converters, the prototype provides an adaptive supply voltage range from 12 V to 100 V to meet the different voltage requirements for various applications. In addition to the adaptive voltage function, direct setting of $V_{DDH}$ via USB is developed in the prototype, providing a programmable voltage function.

### 3.2. High-Precision Current Source

**Figure 4:**
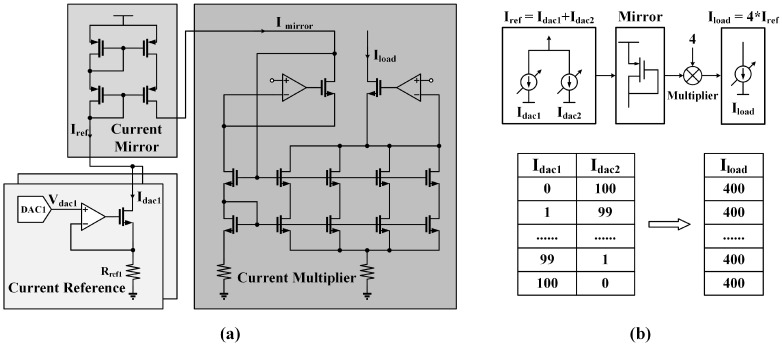
(**a**) Circuit implementation of the high-precision current source block with RDC. $V_{dac1}$ and $V_{dac2}$ generate $I_{dac1}$ and $I_{dac2}$, respectively. $I_{ref}$ is the sum of $I_{dac1}$ and $I_{dac2}$. The current mirror copies $I_{ref}$ to generate $I_{mirror}$, and $I_{mirror}$ is multiplied to obtain $I_{load}$. (**b**) Combining $I_{dac1}$ and $I_{dac2}$ creates a redundant structure that can generate similar output with different inputs.

To improve the current precision, a better approach is to optimize the corresponding digital calibration algorithm instead of simply increasing the circuit complexity. This paper presents a new calibration technique called RDC for improving current accuracy. The proposed algorithm uses a redundant structure that allows different input combinations to generate a same output current. Figure 4a shows the specific circuit design of the high-precision current source block, which is composed of three modules, a current reference, a current mirror, and a current multiplier. The current reference module uses two voltage-controlled current sources to produce two independent currents $I_{dac1}$ and $I_{dac2}$. $I_{ref}$ is the sum of $I_{dac1}$ and $I_{dac2}$, and it can be calculated as

(3) $$I_{ref}=I_{dac1}+I_{dac2}=\frac{V_{dac1}}{R_{ref1}}+\frac{V_{dac2}}{R_{ref2}},$$

where $V_{dac1}$ and $V_{dac2}$ are the output voltages of two commercial DACs, and $R_{ref1}$ and $R_{ref2}$ are the resistances in the current reference module.

$I_{mirror}$ is generated by the current mirror module from $I_{ref}$ and then amplified by a factor of four in the current multiplier to generate output current $I_{load}$. Thus, $I_{load}$ can be presented as

(4) $$I_{load}=4\times I_{mirror}=4\times I_{ref}=4\times \left(I_{dac1}+I_{dac2}\right).$$

As shown in Figure 4b, the outputs of $I_{dac1}$ and $I_{dac2}$ are unbiased under ideal conditions, and different combinations of $I_{dac1}$ and $I_{dac2}$ produce a same $I_{load}$. For example, the following combinations generate a current of 100 least significant bits (LSB), $I_{dac1}$ = 0 LSB and $I_{dac2}$ = 100 LSB, $I_{dac1}$ = 1 LSB and $I_{dac2}$ = 99 LSB, $I_{dac1}$ = 2 LSB and $I_{dac2}$ = 98 LSB, etc. However, $I_{dac}$ is not ideal, and it follows a Gaussian distribution with a standard deviation of σ. When the output current values of $I_{dac1}$ and $I_{dac1}$ are specified to be $I_{1}$ and $I_{2}$, the actual current $I_{dac1}$, $I_{dac2}$, and $I_{load}$ can be calculated as

(5) $$\begin{array}{c} I_{dac1}∼N\left(I_{1},\sigma^{2}\right), \end{array}$$

(6) $$\begin{array}{c} I_{dac2}∼N\left(I_{2},\sigma^{2}\right), \end{array}$$

(7) $$\begin{array}{c} I_{load}∼N\left(4*\left(I_{1}+I_{2}\right),{\left(4\sigma \right)}^{2}\right). \end{array}$$

**Figure 5:**
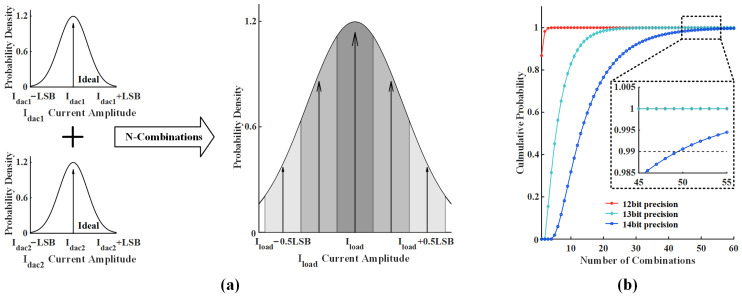
(**a**) With a Gaussian distribution, the actual values of $I_{dac1}$ and $I_{dac2}$ begin to diffuse, which makes achieving higher precision possible by increasing the combination numbers. (**b**) The cumulative probability value of 12-bit, 13-bit, and 14-bit current precision versus the number of combinations indicates that more combinations of $I_{dac1}$ and $I_{dac2}$ are required to obtain higher precision.

Figure 5a shows the probability density functions of $I_{dac1}$, $I_{dac2}$, and $I_{load}$, which indicate the likelihood of obtaining the desired output value. This prototype employs two commercial 12-bit DACs with output codes [0, 1, …, 4094, 4095] and simulates a realistic environment with σ = LSB/3. In this case, the outputs of $I_{dac1}$ and $I_{dac2}$ include sub-integer codes [0.1, 0.2, …, 4094.8, 4094.9], which provides an opportunity to improve the current accuracy, and the high-precision current can be expressed as

(8) $$\begin{array}{c} 12\mathrm{-bit}:\left[0,\;1,\;\ldots ,\;4094,\;4095\right]\subset I_{loa\mathrm{d-}12\mathrm{bit}}, \end{array}$$

(9) $$\begin{array}{c} 13\mathrm{-bit}:\left[0,\;0.5,\;\ldots ,\;4094.5,\;4095\right]\subset I_{loa\mathrm{d-}13\mathrm{bit}}, \end{array}$$

(10) $$\begin{array}{c} 14\mathrm{-bit}:\left[0,\;0.25,\;\ldots ,\;4094.75,\;4095\right]\subset I_{loa\mathrm{d-}14\mathrm{bit}}, \end{array}$$

where the output codes for 12-bit, 13-bit, and 14-bit current accuracy are $I_{loa\mathrm{d-}12\mathrm{bit}}$, $I_{loa\mathrm{d-}13\mathrm{bit}}$, and $I_{loa\mathrm{d-}14\mathrm{bit}}$, respectively.

To achieve a 14-bit current precision, a set of combinations whose absolute error is less than 0.125 LSB must be found for each $I_{loa\mathrm{d-}14\mathrm{bit}}$. The gray regions in Figure 5a show five output currents with different values, $I_{load}-0.5$ LSB, $I_{load}-0.25$ LSB, $I_{load}$, $I_{load}+0.25$ LSB, and $I_{load}+0.5$ LSB. All of the five output currents with an absolute error less than 0.125 LSB can be obtained by increasing the combination numbers *n* of $I_{dac1}$ and $I_{dac2}$, while the $I_{load}$ can be any integer code of [0, 1, …, 4094, 4095]. Thus, the cumulative distribution function $F_{n}$ of obtaining the five desired output currents is

(11) $$F_{n}=\int_{I_{1}+I_{2}+\left(2n-7\right)*\mathrm{LSB}/8}^{I_{1}+I_{2}+\left(2n-5\right)\times \mathrm{LSB}/8}I_{load}*dI,\;\;\left(n=1,2,3,4,5\right)$$

where $F_{n}$ can be obtained by integrating the Gaussian distribution of $I_{load}$.

For *n* combinations, the probability of achieving the 14-bit current accuracy $F_{14\mathrm{bit}}$ is calculated as

(12) $$\begin{array}{c} F_{14\mathrm{bit}}=S\times C\times F, \end{array}$$

(13) $$\begin{array}{c} S=\sum_{n_{1}=1}^{n}\sum_{n_{2}=1}^{n-n_{1}}\sum_{n_{3}=1}^{n-(\sum_{i=1}^{2}n_{i})}\sum_{n_{4}=1}^{n-(\sum_{i=1}^{3}n_{i})}\sum_{n_{5}=1}^{n-(\sum_{i=1}^{4}n_{i})}, \end{array}$$

(14) $$\begin{array}{c} C=C_{n}^{n_{1}}C_{n-n_{1}}^{n_{2}}C_{n-\sum_{i=1}^{2}n_{i}}^{n_{3}}C_{n-\sum_{i=1}^{3}n_{i}}^{n_{4}}C_{n-\sum_{i=1}^{4}n_{i}}^{n_{5}}, \end{array}$$

(15) $$\begin{array}{c} F=F_{1}^{n_{1}}F_{2}^{n_{2}}F_{3}^{n_{3}}F_{4}^{n_{4}}F_{5}^{n_{5}}{\left(1-\sum_{i=1}^{5}F_{i}\right)}^{n-\sum_{i=1}^{5}n_{i}}, \end{array}$$

where *S*, *C*, and *F* are the temporary variables for calculating $F_{14\mathrm{bit}}$.

The cumulative probability curves ($F_{12\mathrm{bit}}$, $F_{13\mathrm{bit}}$, and $F_{14\mathrm{bit}}$) of the combinations *n* to achieve 12-bit, 13-bit, and 14-bit precision are shown in Figure 5b. It can be seen that more redundant input combinations are required to achieve a higher current precision. To obtain a 14-bit current precision with 99% probability, at least 50 sets of redundant combinations are demanded. This work uses the above algorithm to reduce the algorithm complexity in RXF [17] from $\Theta (n^{2})$ to Θ(n), thus achieving a high-precision current source without excessive circuit area and complex calibration algorithms.

### 3.3. Flexible Waveform Generator

After analyzing the theory of the high-precision current source block, RDC is used to improve the current accuracy. To achieve a 14-bit precision, 50 sets of combinations are input for each code in $I_{load-14\mathrm{bit}}$. The maximum combination value is taken if feasible combinations are less than 50. The output codes [0, 0.25, …, 4094.75, 4095] are then obtained by analyzing the measurement results in the external controller, and they should be stored in the look-up table (LUT). This process is performed only once before the first stimuli.

**Figure 6:**
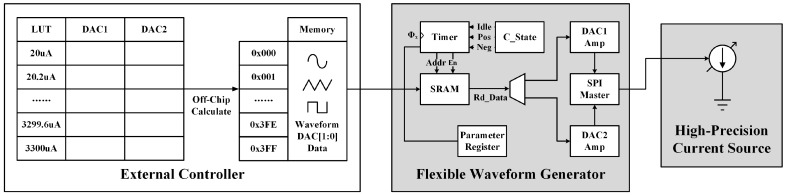
Control strategy of the proposed flexible waveform generator, including one-time off-chip calculation and real-time control realized on FPGA.

As shown in Figure 6, flexible waveforms based on the high-precision current source block can be generated after calibration. Firstly, the current amplitude and waveform type are chosen, such as sine, triangle, rectangle, etc. Second, a one-cycle sample of the desired waveform is taken with more sampling points for smaller distortion. Thirdly, the current values at each sampling point are transformed to a combination of $I_{dac1}$ and $I_{dac2}$ using the LUT, while the combinations should be stored in the memory. After the prototype has been powered on, the data in the memory are written to the static random access memory (SRAM) of the flexible waveform generator. Then, the timer generates the address and enables signals to read the configuration values, while the serial peripheral interface (SPI) master updates the current waveform by adjusting $I_{dac1}$ and $I_{dac2}$. With these control functions, the prototype can perform flexible waveform configurations with high current precision.

### 3.4. HV Electrode Switch Matrix

**Figure 7:**
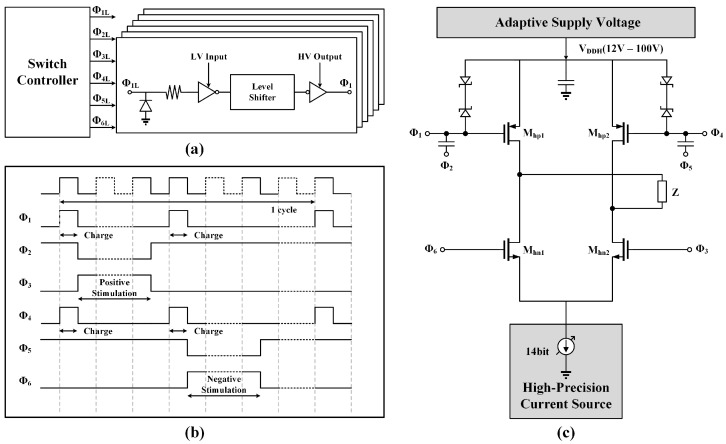
(**a**) Structure of the switch controller and level shifter module. $\Phi_{1L}$, $\Phi_{2L}$, $\Phi_{3L}$, $\Phi_{4L}$, $\Phi_{5L}$, and $\Phi_{6L}$ are the LV control signals generated by switch controller. Level shifters are used to boost the LV control signals and generates HV control signals $\Phi_{1}$, $\Phi_{2}$, $\Phi_{3}$, $\Phi_{4}$, $\Phi_{5}$, and $\Phi_{6}$. (**b**) The timing diagram of switch controller. (**c**) Circuit implementation of HV electrode switch matrix. $M_{hp1}$ and $M_{hp2}$ are the HV PMOS, while $M_{hn1}$ and $M_{hn2}$ are the HV NMOS.

Figure 7c depicts the structure of the HV electrode switch matrix used in this work. The $V_{DDH}$ is provided by the adaptive supply voltage block, and the 14-bit stimulation current is controlled by the high-precision current source block. To control the HV CMOS switches, the voltage value of low-voltage (LV) digital control signals should be boosted by the level shifters.

The system uses 3.3 V-to-12 V HV level shifters to generate control signals $\Phi_{2}$, $\Phi_{3}$, $\Phi_{5}$, and $\Phi_{6}$, while 3.3 V-to-$V_{DDH}$ phototransistors are used to generate control signals $\Phi_{1}$ and $\Phi_{4}$. To protect $M_{hp1}$ and $M_{hp2}$, four reverse Schottky diodes are used to ensure that the $V_{gs}$ of $M_{hp1}$ and $M_{hp2}$ is always within a safe range.

Figure 7b shows the timing diagrams of the boosted control signals. The switches in the HV electrode switch matrix include $M_{hp1}$, $M_{hn1}$, $M_{hp2}$, and $M_{hn2}$. In the charge phase, the voltages of $\Phi_{1}$, $\Phi_{2}$, $\Phi_{3}$, $\Phi_{4}$, $\Phi_{5}$, and $\Phi_{6}$ are $V_{DDH}$, 12 V, 0 V, $V_{DDH}$, 12 V, and 0 V, respectively. All of the switches are in the stable-off state, and no stimulation is output.

When the designed stimulator enters an anodic stimulation phase, the voltages of $\Phi_{1}$, $\Phi_{2}$, and $\Phi_{3}$ are $V_{DDH}$−12 V, 0 V, and 12 V, respectively, while $\Phi_{4}$, $\Phi_{5}$, and $\Phi_{6}$ remain unchanged. At this moment, the switches $M_{hp1}$ and $M_{hn2}$ are turned on, and the stimulator produces an anodic stimulation current. Conversely, the switches $M_{hp2}$ and $M_{hn1}$ are activated, and cathodic stimulation is performed during the cathodic phase. A high-precision flexible stimulation current can be achieved with the techniques mentioned above.

### 3.5. Digital Control

**Figure 8:**
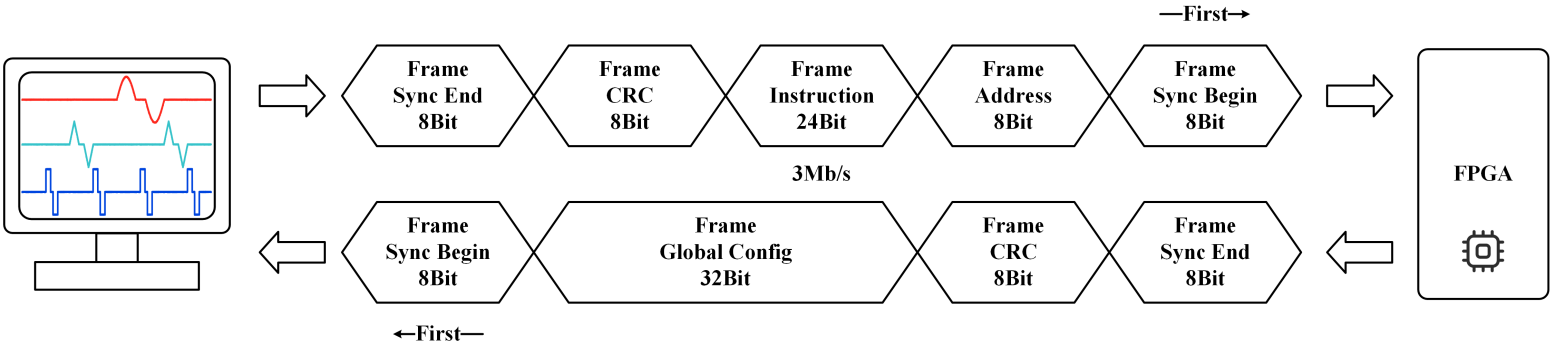
Formats of the forward and backward data frames, which are sent and received via USB at 3 Mb/s.

In the prototype, all digital circuits are implemented based on the FPGA, which receives instructions via a USB interface. Figure 8 illustrates the 8-byte instruction format, including a 1-byte synchronous start signal, a 1-byte address data, a 3-byte configuration data, a 1-byte check value, and a 1-byte synchronous finish signal. The receiver enable function is controlled by the synchronous start and finish signals, while the check value enables the FPGA to ascertain errors in transmission. The corresponding command location is determined by the 1-byte address data, and the instruction content is controlled by the 3-byte configuration data. To properly control the prototype, external devices must follow the specific command formats.

The supply voltage controller is automatically adjusted after the FSM receives an adaptive voltage enable instruction. During stimulation, the FSM also manages the switch controller to choose the stimulation channels and the current direction. The timer in the flexible waveform generator is periodically triggered to produce the current configuration codes from the SRAM. Meanwhile, the high-precision current source block is controlled to output a flexible stimulus waveform.

## 4. Circuit Measurement

**Figure 9:**
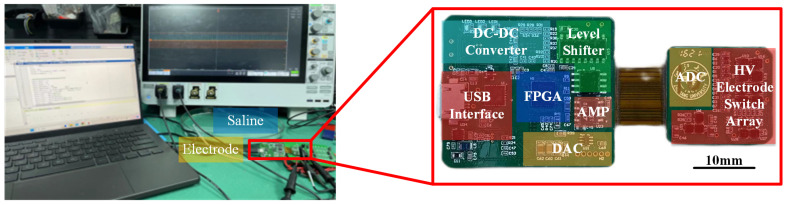
Experiment setup illustration of the designed electrical stimulator prototype. The prototype is controlled by a Matlab program, and the output of the prototype is connected to an electrode array.

Figure 9 shows the measurement setup of the designed prototype, which is connected to a laptop via a USB interface and controlled by a Matlab program. The output of the prototype is connected to an electrode array to perform electrical stimulation. The electrical stimulator was verified through a series of bench-top testings, including a circuit measurement and performance verification in saline.

Table 2 compares the performance of the designed prototype with other state-of-the-art works. The prototype focuses on developing adaptive super-wide supply voltage, high-precision current, and flexible stimulation waveforms. The measurement results are described in the following subsections.

### 4.1. Adaptive Supply Voltage Measurement Results

**Figure 10:**
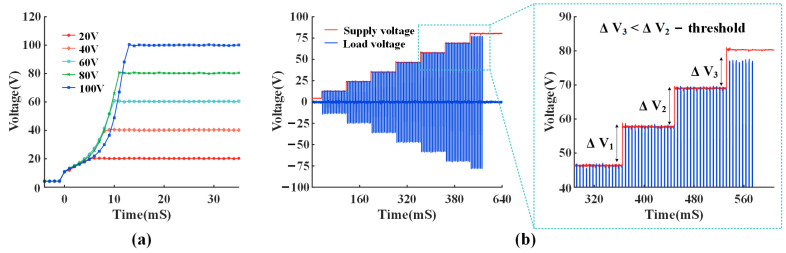
(**a**) Programmable supply voltage controlled directly by manual control. (**b**) Adaptive supply voltage controlled by over-voltage detection algorithm.

This prototype provides two approaches to adjust the HV supply voltage $V_{DDH}$. Figure 10a shows the measurement results of setting $V_{DDH}$ directly, while the adaptive supply voltage block can generate an output voltage from 12 V to 100 V. Five typical amplitudes of $V_{DDH}$ (20 V, 40 V, 60 V, 80 V, and 100 V) are shown with a step of 20 V. The boost time depends on the desired supply voltage, and the maximum value is 12 mS. Figure 10b presents the measured $V_{DDH}$ with the over-voltage detection algorithm. To relieve the discomfort caused by the rapid rise of the $V_{DDH}$ in the electrical stimulator, the algorithm is designed to increase $V_{DDH}$ step by step. An 12-bit ADC is used to monitor $V_{samp}$ to check whether it enters the over-voltage phase when $V_{DDH}$ rises gradually. At the time of 580 mS, a much smaller increase in $V_{samp}$ is detected, which means the current $V_{DDH}$ is the desired supply voltage. Therefore, the over-voltage detection algorithm remains unchanged as $V_{DDH}$ and exits the adaptive adjustment process. If the load is small, a stimulation voltage of 12 V is sufficient to drive the stimulation current. Compared to a fixed supply voltage of 100 V, the stimulation power can be lowered by 88%. With this technique, a wide-range adaptive supply voltage can be achieved without external intervention, thus significantly increasing power efficiency.

### 4.2. High-Precision Current Source Measurement Results

**Figure 11:**
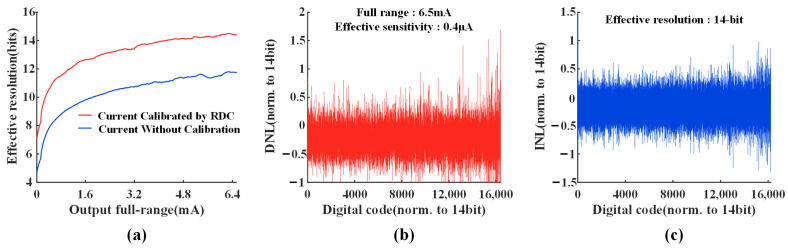
(**a**) Effective current resolution is calculated for each value in one channel. An extra 2-bit current accuracy improvement can be achieved based on RDC. (**b**) Measured differential nonlinearity (DNL) of a stimulation channel with a current range of 6.5 mA and an effective sensitivity of 0.4 μA. (**c**) Measured integral nonlinearity (INL) of a stimulation channel with an effective resolution of 14 bits.

In this work, the resistance of $R_{ref1}$ and $R_{ref2}$ is chosen to be 3.6 KΩ. Under ideal conditions, the maximum output current of $I_{dac1}$ and $I_{dac2}$ is 812.5 μA, while the maximum stimulation current $I_{load}$ reaches 6.5 mA. Figure 11a shows the measured results of the effective resolution, which are defined based on the “Shannon entropy” [17]. Before calibration, the current source is generated by two DACs and influenced by $V_{th}$ and leakage, which will result in significant nonlinearity and lead to a substantial reduction in the current accuracy. After calibration, the proposed RDC technique improves the current resolution by 2 bits, which is consistent with the theoretical analysis. Figure 11b,c illustrate the measured differential nonlinearity (DNL) and integral nonlinearity (INL) of one stimulation channel with RDC. Both the X-axis and Y-axis are normalized to the targeted resolution of 14 bits. The designed prototype achieves a resolution of 14 bits with a full range of 6.5 mA and a sensitivity of 0.4 μA. Due to the limited redundant combinations, the INL and DNL increase locally in higher digital codes, but the overall current accuracy is not affected.

### 4.3. Flexible Waveform Generator Measurement Results

To verify the performance in scenarios with high electrode impedance, the prototype is connected to a 40 KΩ resistor for bench-top testing. The stimulation current adjustment is controlled by the SPI with a delay of 1.6 μS.

**Figure 12:**
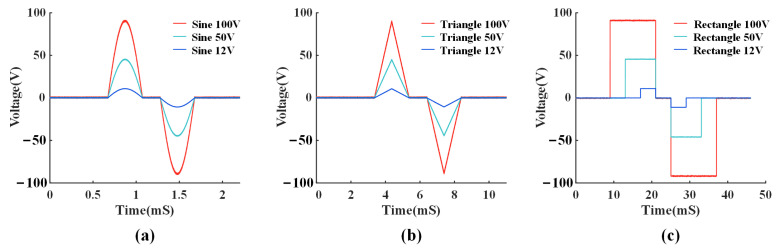
Measured flexible waveforms generated by the prototype on a 40 KΩ resistive load, including (**a**) sinusoidal, (**b**) triangular, and (**c**) rectangular waveforms.

Figure 12 shows the measured flexible stimulation waveforms, including sinusoidal, triangular, and rectangular waveforms with a time precision of 1.6 μS, 5 μS, and 20 μS, respectively. The desired voltage amplitudes of 12 V, 50 V, and 100 V can be obtained by adjusting the $V_{DDH}$.

### 4.4. Measurement Results in Saline

**Figure 13:**
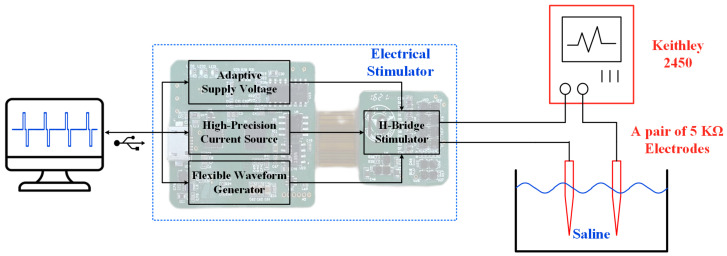
The charge balance-testing procedure. After connecting the device, soak the electrodes in saline and set the stimulation waveform via USB.

The charge balance performance of the designed prototype was verified in saline, and the test process is shown in the Figure 13. The stimulation waveform is an anode-first, bidirectionally symmetrical rectangular stimuli with a pulse width of 2 mS and a current amplitude of 500 μA. The stimulation frequency is 50 Hz, and no special charge balance technique is used in the design. Meanwhile, two steel needle electrodes with an impedance of 5 KΩ at 10 KHz are used to deliver the stimulus. Therefore, the measure of the charge balance performance mainly depends on the current accuracy.

**Figure 14:**
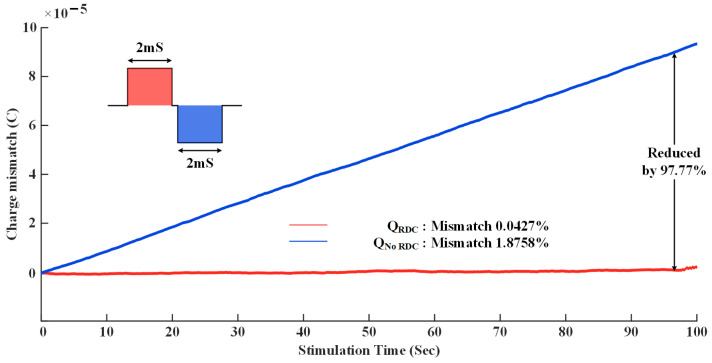
Measured charge balance performance in saline. 97.72% of residual charge is eliminated by employing RDC.

Figure 14 shows the measurement results after long-term stimulation, where high-precision source measure unit (SMU) equipment (Keithley 2450, Tektronix Inc., Beaverton, OR, USA) is used to monitor the stimulation current in real time. The charge mismatch is calculated as

(16) $$\begin{array}{c} Q_{i}=\sum_{i=1}^{i=n}I_{i}\times \Delta T, \end{array}$$

where $Q_{i}$ is the residual charge at time *i*, $I_{i}$ is the amplitude of the stimulation current, and ΔT is 400 μS since the sampling rate of the SMU is set at 2.5 KHz.

The blue curve in Figure 14 gives a charge balance-testing result without any calibration. The current mismatch between anodic and cathodic pulses is 1.8758%, and the residual average DC current is 937.9 nA. The red curve in Figure 14 shows the charge balance performance calibrated by RDC. The current mismatch drops to 0.0427%, and the residual average DC current decreases to 21.35 nA. Through the comparison of the measured charge balance performance before and after calibration, it is shown that 97.77% of the residual charge is eliminated by the proposed RDC algorithm. The designed prototype is supposed to be a safer electrical stimulator structure for long-term stimulation, which is suitable for both implantable and wearable devices.

## 5. Conclusions

A high-precision, super-wide voltage compliant electrical stimulator is designed for neuromodulation. To ensure long-term stimulation safety and achieve arbitrary stimulation parameters under various electrode impedance conditions, several performance improvement techniques are developed in the designed prototype, including over-voltage detection algorithm, redundant digital calibration, and flexible waveform configuration. In the prototype, a super-wide supply voltage range from 12 V to 100 V can be adjusted automatically to realize significant power saving, while an extra 2-bit current accuracy improvement can be achieved without costing too much circuit area. Overall, the proposed prototype can provide adaptive supply voltage, high-precision current, and flexible stimulation waveforms, which are well demonstrated in bench-top testings. Therefore, the prototype is supposed to be a competitive structure for both implantable and wearable applications.

In the future, an application specific integrated circuit (ASIC) based on the aforementioned techniques will be developed to achieve further improvements in power optimization, area saving, and performance enhancement. To better understand the operation mechanism of the brain, neural recording is demanded to realize a closed-loop neuromodulation system.

## Figures and Tables

**Table 1:** Commercial discrete components list.

Components	Company	Model
FPGA	Microsemi	AGLN250-CS81
ADC	ADI	AD7091
DAC	ADI	AD5621
OPAMP	ADI	AD8221
DC-DC ($V_{DDL}$->$V_{DDT}$)	ADI	LT8335
DC-DC ($V_{DDT}$->$V_{DDH}$)	ADI	LT8331
HV PMOS	ON Semiconductor	FDMA86265P
HV NMOS	ON Semiconductor	FDMA86251
HV level shifter	ON Semiconductor	MC14504B
Phototransistor	BROADCO	ACPL-227
USB Interface	FTDI	FT230XQ

**Table 2:** Performance summary and comparison of the designed electrical stimulator.

Parameter	Crunkhorn-2019 [48]	Kassiri-2019 [40]	Nguyen-2021 [17]	Liu-2022 [49]	This Work
Electrical Channels	4	8	8	64	2
Max $V_{DD}$	12 V	24 V	10 V	12 V	100 V
Adaptive Supply Voltage	NO	YES	NO	NO	YES
Max Stimulation Power Saving	N/A	70%	N/A	N/A	88%
Max Current	5 mA	10 mA	1.1 mA	16 mA	6.5 mA
Current Resolution	8-bit	10-bit	9.75-bit	10-bit	14-bit
Charge Balance	Current Source Reuse	NA	Redundant Crossfire	Parameter Control	Redundant Digital Calibration
Flexible Waveform	YES	YES	NO	YES	YES
Time Precision	15.6 μS	100 μS	100 nS	10 μS	1.6 μS

## Data Availability

The data sets generated and analyzed during the current study are not publicly available but are available from the corresponding author upon reasonable request.
